# A novel germline *MLH1* mutation causing Lynch Syndrome in patients from the Republic of Macedonia

**DOI:** 10.3325/cmj.2012.53.496

**Published:** 2012-10

**Authors:** Marija Hiljadnikova-Bajro, Toni Josifovski, Milco Panovski, Aleksandar J. Dimovski

**Affiliations:** 1 Faculty of Pharmacy, Ss. Cyril and Methodius University, Skopje, Republic of Macedonia; 2 University Clinic for Digestive Surgery, Medical Faculty, Ss. Cyril and Methodius University, Skopje, Republic of Macedonia; 3 Clinical Hospital Sistina, Skopje, Republic of Macedonia

## Abstract

**Aim:**

To implement molecular analysis in the clinical diagnosis and management of Lynch syndrome (LS).

**Methods:**

We analyzed the mutations in *MLH1* and *MSH2* in the selected LS families from the Republic of Macedonia.

**Results:**

We performed the very first genetic identification of LS families and characterized a novel mutation. The novel nonsense germline point mutation c.392C>G in the codon 131 of *MLH1*(S131X) was identified as the underlying genetic cause of LS in three families. The haplotype analysis suggested a founder effect of this mutation in our population.

**Conclusion:**

We expect to detect the mutation in other LS patients from the region, and recommend cost-effective screening for this mutation by restriction fragment length polymorphism-polymerase chain reaction or DNA sequencing of *MLH1* Exon5 prior to full genetic testing in all LS suspects of Macedonian ancestry.

Lynch syndrome (LS), formerly known as hereditary non-polyposis colorectal cancer (HNPCC), is considered the most common form of hereditary colorectal cancer (1). It is an autosomal dominantly inherited predisposition to early development of colorectal cancers (CRC), as well as malignancies affecting the endometrium, ovaries, stomach, small intestine, hepatobiliary, and urinary tracts. The genetic foundation of this syndrome in 50%-70% of cases are germline mutations in the mismatch repair (MMR) genes, leading to microsatellite instability (MSI) in the affected tissue as a molecular hallmark of the disease. Germline mutations in *MLH1* and *MSH2* account for 90% of these mutations, but there is evidence for the involvement of *PMS2* and *MSH6* (2-8).

The risk of colon cancer development in a patient with LS before the age of 70 is 52%-82%, ie, 9.5-15 times higher than in the general population (9). Genetic identification of a patient with LS alerts relatives to cancer risk and enables subsequent genetic testing, with a great benefit in terms of timing, expense, and effectiveness of surveillance (10), early detection of polyps, and reduction of cancer mortality. Furthermore, it spares the “healthy” relatives from unnecessary fear and intensified surveillance. Therefore, the evaluation of inherited susceptibility to colorectal cancer is becoming a very common diagnostic and even prognostic tool in colorectal cancer management. Up to now, there have been no official published data on the Lynch Syndrome and genetic testing for familial colorectal cancer predisposition in the Republic of Macedonia. In this regard, our aim was to implement molecular analyses in the clinical diagnosis and management of the LS in the country.

## Materials and methods

This is an ongoing prospective study investigating hereditary colorectal cancer in the Republic of Macedonia. DNA was isolated using Proteinase K digestion/phenol/chlorophorm extraction (11) from fresh tumor tissue and peripheral blood of patients undergoing colon and/or rectum resection at the University Clinic for Digestive Surgery in Skopje. All participants provided informed consent. The research protocol was approved by the Ethics Committee of the Faculty of Pharmacy in Skopje. It included screening for MSI in all tumor tissues, testing for BRAFV600E mutation and promoter hypermethylation of MMR genes in microsatellite instable cancers, followed by germline MMR testing of patients fulfilling Amsterdam II criteria (12) (CRC or LS-related cancers in three or more relatives, one of whom was a first relative to the other two, the cancer was diagnosed in two or more successive generations, at least one of the cases was diagnosed before 50 years, the tumors had to be pathohystologically verified and familial adenomatous polyposis excluded) or revised Bethesda criteria (13) for LS (CRC diagnosed before the age of 50 years, or presence of synchronous, metachronous CRCs or other LS-associated tumors regardless of age, or CRC with the MSI-high histology diagnosed in a patient younger than 60 years, or CRC diagnosed in a patient with one or more first-degree relatives with an LS-related tumor and at least one of whom was diagnosed under age of 50 years, or CRC diagnosed in two or more first or second degree relatives with LS-related tumor, regardless of age).

Paired tumor and blood DNA were used for the evaluation of microsatellite instability applying fluorescent multiplex polymerase chain recation (PCR) of five mono- and dinucleotide markers: BAT25, BAT26, D2S123, D5S346, and D17S250, recommended by the National Cancer Institute (14). PCR-amplified fragments were separated with a capillary gel electrophoresis on AbiPrism310 (Applied Biosystems, Foster City, CA, USA) and a fragment analysis was performed using GeneMapper Software version 4.0.

Multiplex ligation-dependent probe amplification (MLPA) analysis with reagent kits from MRC-Holland (Amsterdam, the Netherlands), based on the technique described by Schouten et al (15) was performed for the detection of large genomic rearrangements in *MLH1, MSH2, MSH6,* and *PMS2*. Methylation-specific MLPA (MS-MLPA) analysis was done with ME011 reagent kit from the same manufacturer for the evaluation of tumor promoter methylation of MMR genes according to the recommendations by Perez-Carbonell (16).

Bi-directional DNA sequencing for the detection of point mutations, small deletions/duplications in *MLH1* and *MSH2,* as well as detection of BRAFV600E mutation, was performed using BigDye Terminator v1.1 Cycle Sequencing Kit reagents (Applied Biosystems) followed by a capillary gel electrophoresis and sequence analysis on AbiPrism 310 genetic analyzer using Sequencing Analysis 5.3.1 computer software (Applied Biosystems).

The haplotype analysis of chromosome 3p was performed on the two probands (index patients), by fragment analysis using fluorescent PCR/capillary gel electrophoresis with four highly polymorphic microsatellite markers: D3S1611, D3S1260, D3S1561, D3S1266 (17). This fragment analysis was also performed on tumor DNA samples of the probands to evaluate the loss of heterozigosity. A restriction fragment length polymorphism-polymerase chain reaction (RFLP-PCR) assay previously described for the detection of the neighbor mutation D132H (18) was employed in screening for the mutation c.392C>G among CRC patients and the control group. Primer sequences and cycling conditions for all of the above PCR reactions are available upon request.****

## Results

The algorithm for the detection of MMR-defects employed in our study was based on MSI-screening of all colorectal cancers regardless of the family history and age at diagnosis, which has already been proven as a cost-effective approach for identifying individuals at risk of LS (19), with a 93% sensitivity of identifying tumors arising from germline MMR mutation (20). This type of genetic instability was identified in 11% (44/389) of the colorectal cancers, selecting them for further genetic evaluation for LS. As tumor *MLH1* promoter methylation and BRAFV600E mutation are proven predictors of a negative MMR mutation status (21-24), we evaluated their status in all microsatellite-instable cancers prior to germline analysis of MMR genes. Using these selection criteria we excluded 26 (59%) patients from futher analysis due to either the presence of the BRAFV600E mutation (5 patients) or hypermethylation of the *MLH1* promoter (21 patients). Finally, 8 patients fulfilling the Amsterdam II or revised Bethesda criteria (12,13) and whose tumors had MSI but lacked both BRAFV600E mutation and MMR genes promoter hypermethylation were selected for laboratory detection of germline MMR defects.

**Figure 1:**
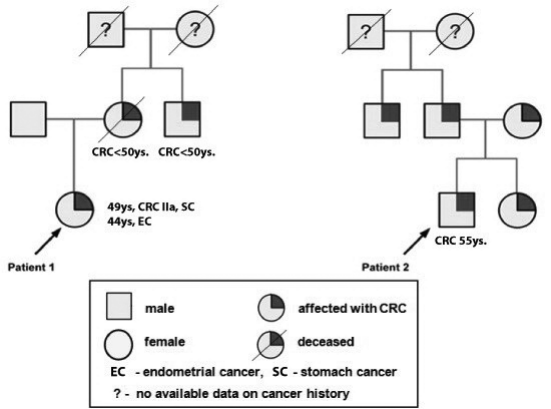
Pedigrees of the two families with Lynch Syndrome.

Following this algorithm, we identified the nonsense point mutation c.392C>G in two patients from different families. Pedigree charts present the patients’ characteristics and show the presence of colorectal cancer in at least two consecutive generations in both families (Figure 1). One of the probands was a woman diagnosed with cancer staged IIa of the transversal colon and a synchronous cancer of the stomach at the age of 49. She had also been diagnosed with endometrial cancer five years before. Her deceased mother and her uncle had also been diagnosed with early onset CRC. The other proband was a man diagnosed with metasynchronous cancers of the transversal and rectosigmoidal colon at the age of 55. His mother, father, uncle, and sister had previously been diagnosed with CRC.

**Figure 2:**
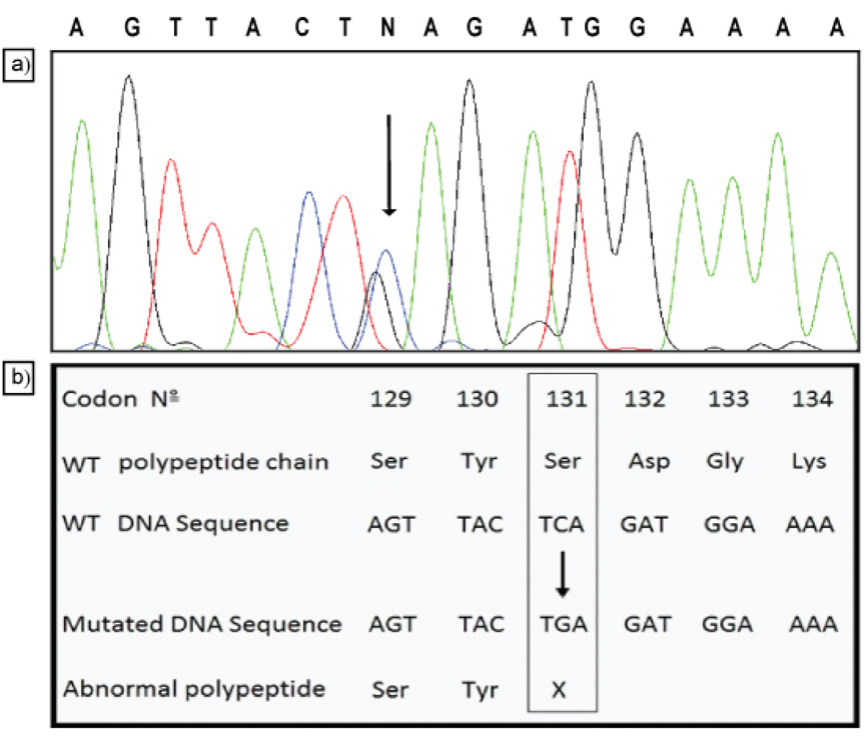
The novel c.392C>G (S131X) mutation underlies the impaired function of *MLH1* detected by microsatellite instability in both Lynch syndrome patients. (**A**) Detection of c. 392C>G mutation by DNA sequencing. (**B**) The C>G conversion turns the wildtype (WT) 131st triplet coding for serine into the TGA (UGA in RNA) termination codon, causing premature end of translation and synthesis of a MLH1 product, which is 626 aminoacids shorter than the wild type protein.

The initial screening for MMR deficiency by MSI testing identified high microstelite instability in the cancers of both probands, and further analysis revealed the absence of BRAFV600E mutation and MMR promoter hypermethylation. Since no large germline deletions or duplications in *MLH1, MSH2, MSH6,* and *PMS2* were detected with the MLPA analysis, blood DNA samples were subjected to the bi-directional DNA sequencing analysis of the 19 *MLH1*- and 16 *MSH2-* exons and flanking regions. The germline point mutation c.392C>G (Figure 2A) was identified in codon 131 of *MLH1* Exon5. The mutation c.392C>G (S131X) caused conversion of the TCA triplet encoding serine to TGA (UGA in RNA), which is a termination codon causing premature end of translation and synthesis of a truncated MLH1 protein consisting of 130 instead of 756 aminoacids (Figure 2B). This mutation can also be detected with a modification of a previously described PCR-RFLP test for the detection of the neighboring D132H mutation. Furthermore, we detected the loss of heterozigosity on chromosome 3p, indicating the loss of the normal allele as the second event of the carcinogenesis in these patients, in accordance with the Knudson’s two-hit hypothesis for cancer development (25). The resulting impaired function of MLH1 is held responsible for the mismatch repair deficiency leading to the development of colorectal cancer with microsatellite instability.

As the probands were not aware of their close common ancestry, we performed a haplotype analysis to evaluate this in more details. The haplotype similarity observed between the two index patients (Table 1) suggested that both patients were descendants of a common ancestor, founder of the c.392C>G mutation but they had either insufficient knowledge of their family trees or the mutation was rather old in the Macedonian population with a possible founder effect. According to these findings, it was understandable to expect identification of the mutation in other LS patients with Macedonian ancestry and therefore we recommend the initial PCR/RFLP testing or *MLH1*Exon 5 sequencing prior to the expensive, full genetic testing in all LS suspects originating from the Republic of Macedonia. Using this approach, we recently detected the same mutation in a third CRC family also without information on close familial relationship with the two previously detected LS families. This finding additionally supports our hypothesis of the founder effect of this mutation in the Macedonian population and justifies the recommendations for the initial screening.

**Table 1:** Haplotypes of the two index patients with the recurring mutation on chromosome 3p. All four of the evaluated microsatellite markers spanning a DNA-region of approximately 10Mb, are present in the genomes of both patients in the same form (length) in either homozygous or heterozygous state

Patient ID	D3S1260[3p22-p21.3] 38454030-38454297*	D3S1611 [3p22.2] 37009261-37009522*	D3S1561[3p22.3] 36424996-36425221*	D3S1266 [3p23] 27899584-27899880*
Patient 1	**260 bp/262 bp^†‡^**	**254 bp/254 bp**	234 bp/**240 bp**	289 bp/**293 bp**
Patient 2	**260 bp/262 bp**	**254 bp**/258 bp	**240 bp**/242 bp	**293 bp**/297 bp

*Position within Homo sapiens chromosome 3 alternate assembly HuRef whole genome shotgun sequence; according to the UniSTS database (26).

†The bolded values denote the common alleles in both patients and point to the mutual haplotype associated with the identified genetic defect.

‡Base pairs (bp).

## Discussion

It is widely documented that colorectal cancer is a major health burden, being the third most frequent type of cancer worldwide, with estimated annual incidence and mortality for 2008 of 1.2 and 0.6 million cases respectively (27). The extended lifespan and the anticipated growth and aging of the human population are expected to increase the number of people with a cancer history in the following years (28). The official CRC-statistics for the Republic of Macedonia are somewhat lower, probably mostly due to misreporting of the cause of death and lack of a unique national register of patients diagnosed with this type of malignancy. The situation is even worse regarding the hereditary syndromes, and until recently diagnosing of familial colorectal cancer was based exclusively on clinical findings and familial history of CRC. Our efforts in establishing and implementing molecular analyses in the clinical diagnosis and management of the disease resulted in the first genetic identification of LS families in the country and enabled the implementation of family members’ genetic counseling.

Our search through The Human Gene Mutation Database (29) revealed that the mutation detected in our patients (c.392C>G) had not previously been identified and reported in other populations. Kurzawski et al (30) have reported a mutation in codon 131 with the same consequence on the protein level S131X, but with a different change in the DNA sequence (c. 392C>A). 

The genetic analysis of other LS suspects is under way and up to now two additional MMR defects have been identified: a deletion spanning exons 3-12 of *MLH1* in a 16-year-old boy by MLPA analysis and a IVS14-19A>G mutation in another patient by DNA sequencing of *MLH1*, which should be functionally analyzed to confirm its involvement in LS. We expect that the results from this study will encourage the establishment of fortified screening programs in the country and region, combining clinicopathological and molecular approaches for identifying families with hereditary CRC syndromes and the underlying genetic cause, as essential steps toward improved prevention of cancer development and reduction of mortality in these families.

One of the index patients had previously been diagnosed with endometrial cancer, but no genetic tests for hereditary syndromes had been performed. Consequently, the LS had not been diagnosed at that point despite the clinical findings and family history of colon cancer. This diagnostic failure may have contributed to the development of the subsequent stomach and colon cancers, which might have been prevented or at least detected in an earlier stage utilizing regular surveillance programs for LS patients. This fact emphasizes the necessity of employing genetic tests in risk assessment for LS in patients with CRC as well as all malignancies related to this syndrome fulfilling the Amsterdam II (12) or revised Bethesda criteria (13).

In conclusion, the very first molecular identification of LS in the Republic of Macedonia revealed a novel mutation in *MLH1*, as a genetic cause for this syndrome in three families unaware of a close common ancestry. Suspecting the founder effect of this mutation, we recommend a routine screening by applying a RFLP-PCR test or DNA sequencing of *MLH1* Exon5, as a cost-effective initial step in the genetic testing of all LS suspects with Macedonian ancestry.
